# BowTieBuilder: modeling signal transduction pathways

**DOI:** 10.1186/1752-0509-3-67

**Published:** 2009-06-30

**Authors:** Jochen Supper, Lucía Spangenberg, Hannes Planatscher, Andreas Dräger, Adrian Schröder, Andreas Zell

**Affiliations:** 1 Center for Bioinformatics Tübingen (ZBIT), University of Tübingen, Sand 1, 72076 Tübingen, Germany

## Abstract

**Background:**

Sensory proteins react to changing environmental conditions by transducing signals into the cell. These signals are integrated into core proteins that activate downstream target proteins such as transcription factors (TFs). This structure is referred to as a bow tie, and allows cells to respond appropriately to complex environmental conditions. Understanding this cellular processing of information, from sensory proteins (e.g., cell-surface proteins) to target proteins (e.g., TFs) is important, yet for many processes the signaling pathways remain unknown.

**Results:**

Here, we present BowTieBuilder for inferring signal transduction pathways from multiple source and target proteins. Given protein-protein interaction (PPI) data signaling pathways are assembled without knowledge of the intermediate signaling proteins while maximizing the overall probability of the pathway. To assess the inference quality, BowTieBuilder and three alternative heuristics are applied to several pathways, and the resulting pathways are compared to reference pathways taken from KEGG. In addition, BowTieBuilder is used to infer a signaling pathway of the innate immune response in humans and a signaling pathway that potentially regulates an underlying gene regulatory network.

**Conclusion:**

We show that BowTieBuilder, given multiple source and/or target proteins, infers pathways with satisfactory recall and precision rates and detects the core proteins of each pathway.

## Background

Most signal transduction events are initialized by cell-surface proteins that respond to specific environmental stimuli. When activated these proteins emanate a signaling cascade which involves a series of (de)-phosphorylation events. In many cases such signaling events transduce the signal to transcription factors (TFs), which in turn regulate the expression level of downstream genes. Understanding this cellular processing of information, from the source proteins (e.g., cell-surface proteins) to the target proteins (e.g., TFs), is important when generating comprehensive models of regulatory networks. For several biological processes the signaling pathway has been derived experimentally [1,2]. However, a large number of complex signaling pathways are yet to be discovered. To unravel these, computational inference methods are a valuable tool.

The basis for the computational inference of novel signaling pathways are protein-protein interaction (PPI) datasets. These datasets are derived from biological studies on individual PPIs, but recently also by large-scale genomic, proteomic, and bioinformatic analyses. The yeast-two hybrid method, for instance, was a major driving force in this development [3-5]. These technological advances in measuring and predicting PPIs have fueled numerous databases [6-9].

Based on such PPI datasets several methods have been developed for inferring signal transduction pathways [10-13]. Some of these methods combine PPI with gene expression datasets [10,13], improving the overall performance. Here, the dataset provided by the STRING database is utilized [9]. STRING already integrates PPI information from various sources (e.g., coexpression, the literature, and genomic context) and provides confidence scores for each reported PPI.

**Figure 1:**
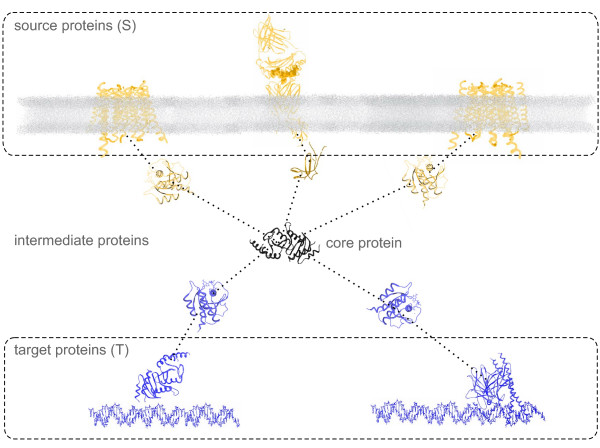
**Assumed structure of signaling pathways**. This figure depicts a signal transduction pathway. The source proteins are cell-surface proteins that transduce the signal to intermediate (cytosolic) proteins. These in turn transduce the signal to the target proteins (TFs), that regulate the transcription of downstream genes. A dotted line between two proteins indicates a PPI.

When inferring signaling pathways some assumptions regarding their structure have to be made. Many previous approaches have inferred pathways by connecting pairs of proteins (e.g., one membrane protein and one TF) [10,13,14]. In recent works on the structural organization of cellular regulation, however, it has been reported that many biological networks are structured like bow ties [15-18]. Such bow tie structures contain multiple source and target proteins and, in most cases, internal proteins that process the transduced signals (Figure 1).

In this work we present BowTieBuilder, which aims at integrating multiple source proteins (e.g., membrane proteins) and target proteins (e.g., TFs) into one signaling pathway. As input, BowTieBuilder requires a set of source and/or target proteins. Given this input, BowTieBuilder searches for the most probable pathway that connects the input and output proteins. Thereby, core proteins are favored implicitly through the objective function. For every inferred signaling pathway, the core proteins are determined and their bow tie score is calculated, this value indicates whether the pathway is bow tie structured. These core proteins constitute gateways that integrate all information and are, therefore, often the key regulators in these signaling pathways. In contrast to metabolic networks where the core often forms a large cluster with interconnected nodes, bow ties in signaling networks are reported to have fewer nodes with sparse interconnections [19] – if they exist at all. Accordingly, The BowTieBuilder does not require a bow tie structure, so that pathways without a bow tie structure can also be inferred and analyzed.

To validate this method various sets of source and target proteins from yeast (*Saccharomyces cerevisiae*) and human (*Homo sapiens*) are inferred and compared against signaling pathways from KEGG (Kyoto Encyclopedia of Genes and Genomes) [20]. To compare BowTieBuilder to other heuristics, three additional inference methods are described and applied to the same pathways. After validating the results against KEGG signaling pathways, two signaling pathways with no related KEGG pathway are inferred. One pathway involved in the innate immune system of human and another pathway that connected signal transduction and gene regulatory networks, which was inferred in a separate study [21].

## Methods

### Protein-protein interaction (PPI) data

The PPI dataset is represented as a weighted directed graph *G *= (*V*, *E*, **w**), where nodes (*V*) represent proteins, edges (*E*) PPIs, and the scores (**w**) the confidence in each interaction. The scores (edge weight **w**) range from 0, indicating no interaction, to 1, indicating an interaction with high confidence.

The PPI dataset used in this work is obtained from the STRING database (version 7.1) [9,22-24]. This dataset contains computationally and experimentally derived PPIs, including interactions from other databases (e.g., MINT [25], BioGRID [26], DIP [27], and Reactome [28]), microarray experiments, high-throughput experiments, and a mined literature corpus. Furthermore, PPIs are transferred between orthologous pairs of proteins over different organisms. All of these datasets are combined and for each PPI a confidence score is calculated. This way the information from multiple sources is combined into a single score that expresses the overall confidence in each PPI. This score is derived by calculating the joint membership of proteins with PPI in KEGG pathways [29].

### Problem complexity and formalization

The problem posed here is similar to the problem of finding Steiner trees in graphs [30], or more specifically, vertex-weighted Steiner trees. In this problem formalization, a weighted graph *G *= (*V*, *E*, **w**) and a non-emtpy set of terminals *T *⊆ *V *is given, with **w **∈ ℝ^+^. The optimal Steiner tree is defined as the connected subgraph *G' *= (*V'*, *E'*, **w'**) with *G' *⊆ *G*, for which the summed weight **w**_sum_(*E'*) = ∑_**e**∈*E' *_**w**_*e *_is minimal, and *T *⊆ *V' *holds. The Steiner tree problem on graphs was shown to be -complete [31] and, thus, is in most cases solved with heuristics. One of these heuristics is Prim's algorithm [32], which iteratively extends the subgraph *G*' by adding the vertex with the smallest distance until all nodes in *T *are connected in *G*'. A more recent heuristic presented by Melhorn *et al*. [33] proceeds by first calculating the minimal distance between all nodes in *T*, and then assembling the minimal Steiner tree by iteratively connecting the nodes with the smallest distance to each other.

Here, the aim is to select a subgraph of *G' *⊆ *G *that connects a set of source proteins *S *to a set of target proteins *T*. Given a graph *G *= (*V*, *E*, **w**) with *w *∈ [0, 1] and a disjoint source *S *⊆ *V *and target set *T *⊆ *V *(*S *∩ *T *= ∅), the aim is to find the optimal subgraph *G' *⊆ *G *such that for every *s *∈ *S *and for every *t *∈ *T *at least one path *P*(*s*, *t*) exists in *G'*, whenever such a path exists in *G*. If either the source or the target set is empty, the problem formalization of Steiner trees is applied. Then the aim is to connect all nodes that are given either in *S *or in *T *(*S *∩ *T*) to each other.

### Objective function for the pathways

For any given pathway, the overall confidence is calculated by multiplying the individual confidence values of the utilized edges:

(1)

This objective is based on the assumption that the edge scores reflect independent confidence values, and implies that the resulting score gives the overall confidence in the pathway – that all contained edges are true biological interactions.

### Inferring signal transduction pathways

#### Finding optimal paths between two proteins

Although the problem of finding the optimal pathway is -complete, some special instances exist that are solvable in polynomial time. If, for instance, the source set *S *and target set *T *both contain one node, the problem reduces to finding the highest scoring path between them. This problem can be solved by applying Dijkstra's algorithm [34]. Given two nodes, this algorithm finds the highest scoring path with a runtime complexity of ((|*E*| + |*V*|) log |*V*|), where |*V*| gives the number of proteins and |*E*| the number of PPIs. For PPI networks, it can be assumed that most proteins are not connected to each other |*E*| ≪ |*V*|^2^; therefore, Dijkstra's algorithm is implemented using adjacency lists, and thus the runtime is reduced to (|*V*| log |*V*| + *E*). The scores between all nodes, obtained by Dijkstra's algorithm, will be stored in a distance matrix *D*_|*S*|×|*T*| _with |*S*| rows and |*T*| columns and the respective paths will be referred to by *P*^*D*^(*s*, *t*).

#### BowTieBuilder

When multiple source and target proteins are provided, we employ a greedy approach, referred to as BowTieBuilder, to construct the signaling pathway *P*. In the first step, BowTieBuilder initializes the signaling pathway *P *= (*V *= *S *∩ *T*, *E *= ∅, **w **= ∅) by including the source *S *and target *T *nodes, and flagging these nodes as 'not visited'. In the second step, the distance matrix *D*_|*S*|×|*T*| _is constructed by determining the maximal scoring (Equation 1) paths between the nodes in *S *and the nodes in *T *with Dijkstra's algorithm, where the distance is set to ∞ if no path exists. This preprocessing is similar to the heuristic presented by Melhorn *et al*. [33] for finding Steiner trees. In the next stage of the inference, the highest scoring path *P*^*D*^(*s*, *t*) in *D *that connects a 'not visited' node to a 'visited' node is added. If no such path exists the two 'not visited' nodes with the highest scoring path *P*^*D*^(*s*, *t*) in *D *are connected to each other and, likewise, the path *P*^*D*^(*s*, *t*) is added to *P*. Subsequently, the nodes in that path are flagged as 'visited' and *D *is updated to include all distances to the nodes in *P*^*D*^(*s*, *t*). This step is reiterated, in each stage integrating 'not visited' source and target nodes. The method terminates when all nodes in *S *∩*T *are flagged as 'visited', or, if for the remaining nodes, no path to any other node in *S *∩ *T *exists. Then the final signaling pathway *P *is returned. If either *S *or *T *is an empty set, *D *is initialized such that it contains all distances between any node in the input set (*D*_|*S*∩*T*|×|*S*∩*T*|_). Despite this change in the initialization of *D*, the algorithm proceeds in the same manner and finally returns the signaling pathway *P *which connects all nodes to each other. The structure of the BowTieBuilder algorithm is given in the following:

1. Initialize the pathway *P *with all nodes *S *∩ *T*, and flag all nodes in *S *∩ *T *as 'not visited'.

2. Calculate the distance matrix *D*_|*S*|×|*T*| _between the nodes in *S *and *T *with Dijkstra's algorithm.

3. Select the shortest path in *D *that connects a 'not visited' and a 'visited' node in *P*, or, if no such path exists, a 'not visited' node in *S *to a 'not visited' node in *T*.

4. Add the nodes and edges of the selected path to *P *and flag all nodes in the pathway as 'visited'.

5. Update *D *to include all distances to the nodes in *P*^*D*^(*s*, *t*).

6. Repeat the steps 2–5 until every node in *S *is connected to some node in *T*, and vice versa if such a path exists in *G*.

7. Export final pathway *P*.

As an optional parameter, the maximum path length *l *is introduced, since very long paths can increase the introduction of false positive PPIs. This is accomplished by setting the length of a path with more than *l *edges to ∞.

#### Additional inference methods

When applying heuristics, it is advisable to compare different approaches to each other to analyze their properties. For this purpose, we implemented three alternative inference methods: *all interactions*, *shortest paths*, and *all shortest paths*.

***all interactions***: In this modification, the standard BowTieBuilder is applied and the resulting pathway *P *is obtained. Then, all PPIs (edges) between any two nodes in *P *are added whenever they are contained in *G*.

***shortest paths***: In this inference method, every node in the source set *S *is connected to the target set *T *through the maximal scoring path, and vice versa. In this case the pathway *P *can be directly derived from paths corresponding to the maximal scores in matrix *D*. More specifically, for each row and column, the path corresponding to the maximal entry in *D *is added to *P*.

***all shortest paths***: In this inference method, for every pair of source (*S*) and target (*T*) proteins the highest scoring path *P*^*D*^(*s*, *t*) is added to *P*. Thus, every source and target node is directly connected if a corresponding path exists in *G*.

#### Output

Inferred signal transduction pathways are exported in the formats GML (Graph Markup Language), XGML, and GraphViz, and visualized with the graph viewer yED [35] or by Cytoscape [36,37].

### Validation

To validate the correctness of the inferred pathways we compute the recall and precision rates with respect to a specified reference pathway. These rates can be calculated with respect to PPIs or proteins. The recall rate is defined as the fraction of PPIs/proteins in the reference pathway that are inferred (Equation 2) and the precision rate is defined as the fraction of inferred PPIs/proteins that are contained in the reference pathway (Equation 3).

(2)

(3)

The topological validation is only performed for pathways that are provided by KEGG. Another possibility for testing the plausibility of inferred pathways – without the need for validation pathways – is to test if the inferred pathway can be associated with a certain biological process. To perform such an analysis, we map the proteins contained in each pathway to their 'biological process', defined by the Gene Ontology (GO) [38]. The tool Term Finder [39] is used for this purpose, which calculates a *p*-value for each biological process using the hypergeometric distribution.

A direct validation against other methods for automatically inferring signal transduction pathways is omitted, because most of these algorithms are validated through pathways with one source and one target protein [10,11,13]. The recall and precision rates obtained by the different methods can, however, give a rough estimate of the relative performance.

### Source and target proteins

BowTieBuilder is applied to several sets of source and target proteins. In principle, any type of source or target protein can be processed by BowTieBuilder; in this work, however, if not stated otherwise, the source proteins are membrane-bound proteins and the target proteins are TFs.

To infer signaling pathways for different biological processes, we collect several sets of membrane-bound proteins and TFs. To infer signaling pathways that control the yeast cell cycle, we collect membrane-TF sets for the yeast cell cycle phases G1 and S from the respective KEGG pathway (KEGG identifier: sce04111). For the analysis of the yeast MAPK pathway, the membrane and TF sets are obtained from the KEGG MAPK pathway (KEGG identifier: hsa04010). In addition, the human membrane and TF sets of the Erb pathway are collected from KEGG (KEGG identifier: hsa04012), and the human membrane and TF sets related to the TLR-mediated innate immune pathway are collected from a publication of Kitano *et al*. [18].

To combine signal transduction pathways with gene regulatory networks, all TFs that were inferred as regulators in a previous study [21] are used here as the target list. In this study, TFs were inferred to have a regulatory effect from two gene expression datasets [40,41] and known *cis*-regulatory elements. In addition to these TFs a list of membrane proteins was collected from the Yeast Membrane Protein Library (YMPL). Based on these TFs and membrane proteins, a signaling pathway is inferred that potentially explains the higher-level regulation of these TFs in the respective gene regulatory network. All source and target proteins are provided in Additional File 1.

### Bow tie score

As mentioned earlier, BowTieBuilder favors signaling pathways that are structured like a bow tie, but it does not demand such a structure. Thus, it is of interest to quantify to what extent signaling pathways follow the bow tie structure and, in addition, to determine the core proteins. For this purpose, we provide a bow tie score (*b*(*p*) ∈ [0, 1]) that determines how 'central' a protein *p *is. This score is also used to determine the bow tie score of the complete pathway. This score is related to the 'betweenness' measure, in which the number of shortest paths that include the core protein determines the centrality.

To calculate this score, the possible number of connecting paths between the source *S *and target *T *proteins is first determined, which is simply the number of source proteins multiplied with the number of target proteins |*S*|·|*T*|. Then the number of source and target proteins that can be connected by a path containing *p *is calculated. This is given by the number of target proteins from which *p *can be reached (|*T*_*p*_|) multiplied by the number of source proteins that can be reached from *p *(|S_*p*_|). Thereby, every edge can only be traversed in one direction, since the signaling pathway is a directed graph that is traversed from the source to the target proteins. The corresponding bow tie score for any protein *p *reads:

(4)

To determine the core elements of any signaling pathway, *b*(*p*) is calculated for every intermediate protein *p*. Given these scores, the core component is defined by the set of proteins with the maximal *b*(*p*) score. This also gives the overall score of the signaling pathway. In some cases it is helpful to distill the subnetwork that constitutes the bow tie structure by removing all paths that do not pass through the core component. We refer to such signaling pathways as 'core bow tie'.

## Results

### Validation with KEGG signal transduction pathways

To evaluate all heuristics, they are applied to the G1-phase cell cycle, S-phase cell cycle, MAPK pathways of yeast, and the human Erb pathway. The resulting recall and precision rates are provided in Table 1. In comparison to other heuristics, BowTieBuilder has the highest average precision with respect to proteins and PPIs. This could be expected since BowTieBuilder aims at finding the minimal pathway *P*, whereas the other methods add additional PPIs or proteins to the pathway. The *shortest paths *heuristic has the highest protein recall rate, whereas the *all interactions *heuristic has the highest PPI recall rate.

**Table 1:** Comparison of different heuristics.

		BowTieBuilder	shortest paths	all shortest paths	all interactions
G1-Phase	precision (PPI)	77%	77%	77%	48%
	recall (PPI)	77%	77%	77%	77%
	precision (protein)	67%	67%	67%	67%
	recall (protein)	67%	67%	67%	67%

S-Phase	precision (PPI)	73%	70%	59%	60%
	recall (PPI)	55%	60%	50%	56%
	precision (protein)	86%	86%	86%	86%
	recall (protein)	86%	86%	86%	86%

MAPK	precision (PPI)	46%	46%	37%	31%
	recall (PPI)	37%	43%	45%	41%
	precision (protein)	79%	80%	69%	79%
	recall (protein)	74%	78%	80%	74%

Erb	precision (PPI)	55%	40%	9%	NA
	recall (PPI)	28%	25%	15%	NA
	precision (protein)	79%	72%	44%	79%
	recall (protein)	56%	76%	73%	56%

average	precision (PPI)	63%	58%	46%	46%
	recall (PPI)	49%	51%	47%	58%
	precision (protein)	78%	76%	67%	78%
	recall (protein)	71%	77%	77%	71%

Statistical evaluation of signal transduction pathways inferred with BowTieBuilder and alternative heuristics. The inferred signal transduction pathways are mapped against the reference signal transduction pathways from KEGG. The precision and recall rates are calculated with respect to the PPIs and proteins.

Depending on the type of validation (protein or PPI), the performance of some heuristics varies strongly. The *all interactions *heuristic, for instance, has high precision when inferring proteins, although the precision for inferring edges is significantly lower in comparison to BowTieBuilder.

In summary, BowTieBuilder has the highest average precision but the lowest average recall. The *all interactions *heuristic, on the other hand, has the lowest precision and highest recall rate. Thus, the average precision decreases and the average recall increases in the following order: BowTieBuilder, *shortest paths*, *all shortest paths*, and *all interactions*. Several of the inferred pathways are provided in Additional File 2.

### Yeast cell cycle pathways

**Figure 2:**
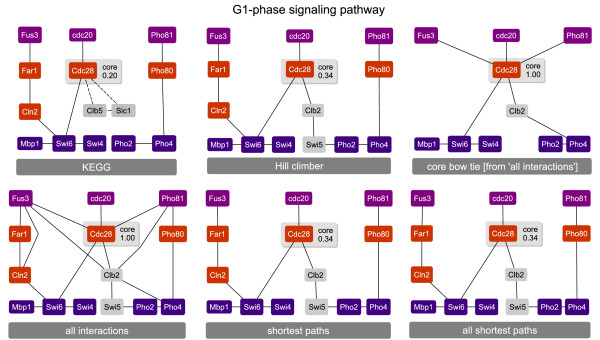
**G1-phase signaling pathways inferred with different heuristics**. The membrane-bound proteins are depicted at the top, the TFs are depicted at the bottom, and the inferred proteins in between (except SWI5 which is an inferred TF). Proteins that occur in the KEGG pathway but are not inferred by any heuristic are depicted in gray. The core protein is CDC28 in all cases – its bow tie score is provided in the respective box. All recall and precision values are provided in Table 1.

For the inferred G1-phase cell cycle pathway, the PPI precision rates range from 48% to 77%, whereas the PPI recall is 77% in all cases (Table 1). The most significant biological process for the inferred proteins is 'cell cycle' (*p*-value: 4.00·10^-5^). Four of six proteins from the KEGG pathway are contained in all inferred pathways (Figure 2), thus the protein recall and precision rates are 67%. The two proteins (Sic1 and Clb5) that constitute alternative paths through the signaling pathways in KEGG are not considered by any inference method. In all cases the core protein is Cdc28, however, its bow tie score ranges from 0.20 (KEGG pathway) to 1.00 ('*all interactions*'). Cdc28 is reported to be the central coordinator of the major events of the yeast cell division cycle [42].

**Figure 3:**
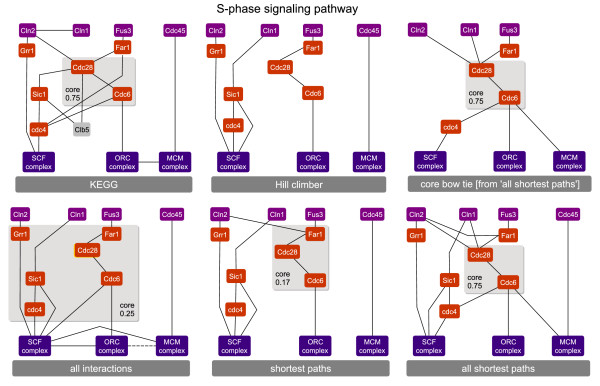
**S-phase signaling pathways inferred with different heuristics**. The membrane-bound proteins are depicted at the top, the TFs are at the bottom and the inferred proteins in the middle. Proteins that occur in the KEGG pathway but not inferred by any heuristic are depicted in gray. The varying core proteins are indicated through gray boxes, which provide their bow tie score. All recall and precision values are provided in Table 1.

In the case of the S-phase pathways, Cdc28 is also contained in all core components, except in the BowTieBuilder pathway which is not bow tie structured. The KEGG core component containing Cdc28 and Cdc6 with a bow tie score of 0.75 is also found in the *'all shortest paths' *pathway (see Figure 3). The inferred S-phase pathways lack only the protein Clb5 in all cases. This protein binds to Cdc28 and Sic1, and thereby introduces a cycle into the signaling pathway. Hence, this protein is not considered by approaches searching for minimal graphs. Accordingly, the protein recall and precision rates are 86% in all cases, whereas the PPI recall and precision rates range from 50% to 73%. The proteins inferred in the case of the S-phase cell cycle show a significant enrichment for 'G1/S transition of mitotic cell cycle' (*p*-value: 9.78·10^-13^).

### Yeast MAPK pathway

**Figure 4:**
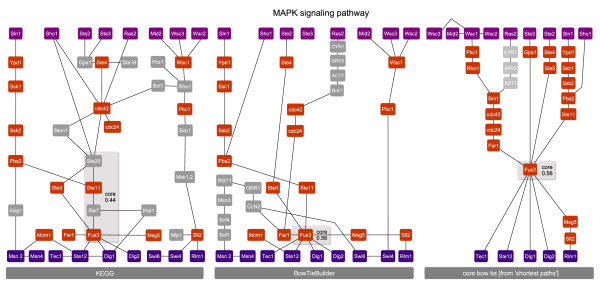
**MAKP signaling pathway of yeast**. Depicted is the KEGG pathway and the pathway inferred by BowTieBuilder. The membrane-bound proteins are drawn at the top, and the TFs are drawn at the bottom. The inferred proteins are depicted in between. Proteins that do not overlap between the KEGG and BowTieBuilder pathways are depicted in gray. The core proteins are embedded in a gray box, where FUS3 is contained in all core structures. The recall and precision values are given in Table 1.

The MAPK pathway inferred with BowTieBuilder contains several 'shortcuts' with respect to the original pathway in KEGG (Figure 4). For instance, the inferred pathway connects FAR1 directly to Cdc24 (STRING score: 0.99) and FUS3 directly to STE11 (STRING score: 0.99), whereas in KEGG they are connected through intermediate proteins. These 'shortcuts' are high-confidence PPIs in STRING and experimentally verified, thus allowing inference of shorter pathways than those given in KEGG. At this point it is unclear which connections are actually utilized in the cell, or even if this utilization depends on the specific environmental conditions. Overall, the PPI recall and precision rates are rather low, whereas the protein recall and precision rates are up to 79% (in the case of BowTieBuilder and the *all interactions *heuristic). The MAPK pathway responds to different external stimuli, such as pheromones and osmolarity. In accordance with these known MAPK stimuli, the GO processes 'osmotic stress' (*p*-value: 2.57·10^-12^) and 'response to pheromone' (*p*-value: 1.36·10^-11^) are the most significant. The core proteins that are contained in the KEGG pathway are: Ste20, Ste11, Ste7 and Fus3. Together with Ste5 these proteins form a scaffolding complex. In the inferred networks this complex is not present because of the '|shortcut' from Ste11 to Fus3. Nonetheless, Fus3, the endpoint of this scaffolding complex, is the core protein in both inferred pathways with a bow tie score similar to the KEGG pathway.

### Human Erb pathway

**Figure 5:**
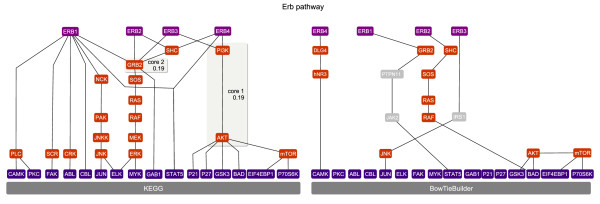
**Erb-associated signaling pathways**. Erb signal transduction pathway inferred with BowTieBuilder. Membrane-bound proteins are depicted at the top, TFs are depicted at the bottom, and the inferred proteins are depicted in between. The core proteins are embedded in a gray box, in which their bow tie score is provided. For the inferred Erb pathway, however, no core element could be determined. The recall and precision values are given in Table 1.

The inferred human Erb pathway is mapped to the GO term 'erb signaling pathway' (*p*-value: 2.51·10^-30^) as the most significant biological process. Thereby, several structures also found in the KEGG pathway could be observed (Figure 5), however, with rather low recall and precision rates (Table 1). Several proteins are skipped by 'shortcut' PPI as already observed for the MAPK pathway. For the inferred Erb pathway no bow tie structure could be found.

### TLR-mediated innate immune pathway

**Figure 6:**
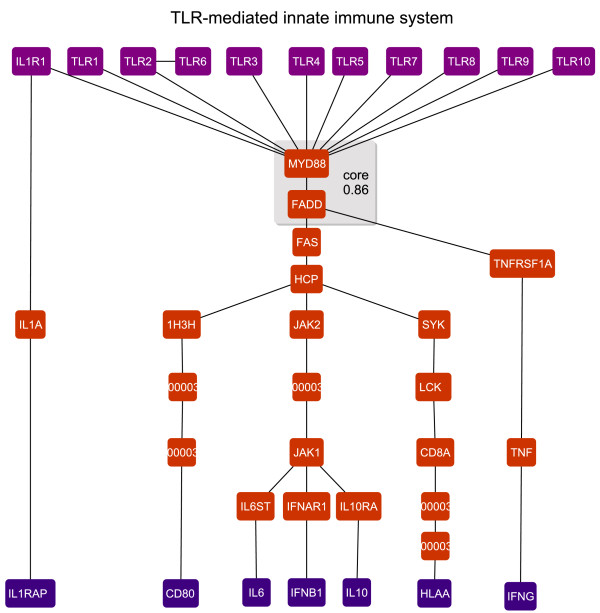
**TLR-mediated innate immune signaling pathway**. TLR-mediated innate immune-associated signaling pathway inferred by BowTieBuilder. Membrane-bound TLR-proteins are depicted at the top, TFs are depicted at the bottom and the inferred proteins are depicted in-between. The proteins MyD88 and FADD are both in the core module and considered to be essential to this signaling pathway.

The TLR-mediated innate immune system of humans is known to have a bow tie architecture in which eleven TLRs respond to a wide variety of pathogens, capturing so-called pathogen-associated molecular patterns. MyD88 is responsible for the activation of TLR-mediated responses. For this pathway no applicable validation pathway is available in KEGG. Nonetheless, the inference of this pathway revealed the general structure of the TLR-mediated innate immune pathway (Figure 6). Furthermore, a clear bow tie structure can be observed with MyD88 and Fadd as core elements, which is also reported in the publication of Oda *et al*. [17].

### Integrating signal transduction pathways and gene regulatory networks

**Figure 7:**
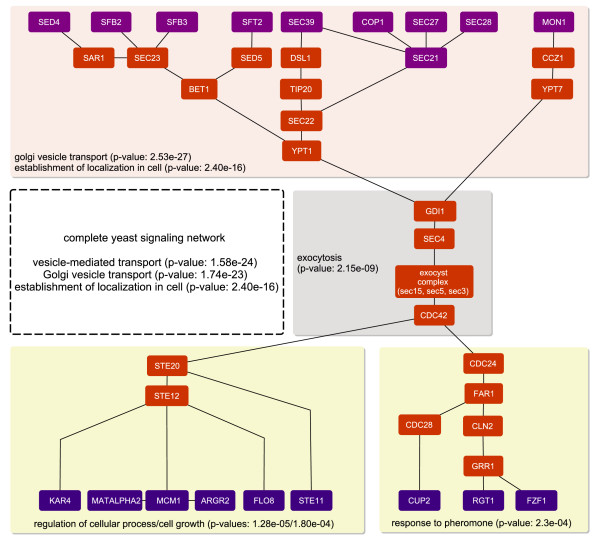
**Signaling pathway inferred from a gene regulatory network**. This figure depicts a bow tie inferred from membrane-bound proteins and TFs that were predicted to be active in a gene regulatory network [21]. The proteins in the bottom are the TFs, the proteins at the top are the membrane-bound proteins and the remaining proteins constitute the inferred signaling pathway. Different modules of this pathway are associated with different biological processes, where the core module is associated with exocytosis – a process through which cells direct secretory vesicles out of the cell.

For a set of TFs obtained by inferring a gene regulatory network [21], BowTieBuilder was applied to infer the corresponding signaling pathway. This inference leds to several distinct signaling pathways, and the one with the highest bow tie score is depicted in Figure 7. The core component of this pathway contains several proteins, including the exocytic complex, that are related to excocytosis. These core proteins connect various membrane-bound proteins and TFs, and can be divided into different subpathways that are related to different biological processes (Figure 7).

## Discussion

In this work, we have presented several heuristics that allow inferring signal transduction pathways when several source and/or target proteins are given. The resulting pathways provide the researcher with a interconnected signaling pathway, which unravels the core proteins that integrate and transduce signals from multiple source to multiple target proteins.

Most current methods for the automated reconstruction of signal transduction pathways infer linear pathways and incorporate gene expression data into their scoring function [10-14]. BowTieBuilder allows inferring signal transduction pathways from an arbitrary number of source and target proteins.

Furthermore, the scoring function of BowTieBuilder is based solely on the PPI dataset from STRING and the associated confidence values. Hence, we build upon the integration of PPI information by STRING. The inferred signaling pathways had satisfactory recall and precision rates for most signaling pathways; for some, however, there is room for improvement. Two main sources of error could be observed. The first source of error was that some PPIs allow a 'shortcut' from the source to the target protein, in comparison to the reference pathway. This was, for instance, the case for several MAPK pathways, where these 'shortcuts' were even interactions with a high confidence level. Another source of error arises from the cyclic patterns that are neglected by inference methods when maximizing the pathway score.

## Conclusion

In conclusion, when keeping the potential pitfalls of such inference methods in mind, the signaling pathways obtained can be of great help in understanding and constructing regulatory networks. BowTieBuider is capable of uncovering core proteins that integrate multiple source proteins and transduce these signals to TFs. This could be observed for the TLR-mediated innate immune pathway, where MyD88 and Fadd constitute the core proteins that function as a hub for all possible signaling pathways. Furthermore, Cdc28 was inferred as a core protein in both cell cycle related pathways, which is confirmed in the literature [42].

In other cases, such as the Erb pathway, no clear bow tie structure could be uncovered. Furthermore, proteins that were core proteins in certain pathways (e.g., Cdc28 in both cell cycle pathways) had very low bow tie scores in other pathways. Thus, which proteins constitute the core of a signal transduction pathway seems to be dynamic and depends on the context of target and source proteins.

## Competing interests

The authors declare that they have no competing interests.

## Authors' contributions

JS wrote the manuscript and conceived this work. LS and JS developed the methods and algorithms. LS implemented the algorithms in Java™. AD, AS, HP, and AZ were involved in the study design and coordination. All authors read and approved the final manuscript. None of the authors have any competing financial or other interests in relation to this work.

## Supplementary Material

Additional file 1 **All target, source, and inferred proteins of the signaling pathways**. All source, target, and inferred proteins are provided in an Excel file, along with their significant GO-terms. Click here for file

Additional file 2 **GraphML-formatted files of several signaling pathways**. Several signaling pathways in GraphML format. These files can be viewed and edited with yEd (from yWorks). Click here for file
